# Supplementary material for: Low-Temperature-Meltable Elastomers Based on Linear Polydimethylsiloxane Chains Alpha, Omega-Terminated with Mesogenic Groups as Physical Crosslinker: A Passive Smart Material with Potential as Viscoelastic Coupling. Part II—Viscoelastic and Rheological Properties
Source: Polymers (Basel). 2020 Nov 29;12(12):2840. doi: 10.3390/polym12122840 (PMC7760245; doi:10.3390/polym12122840)
Supplement: Supplementary file 1 [file polymers-12-02840-s001.pdf]

Low-temperature-meltable elastomers based on linear polydimethylsiloxane chains alpha,omega-terminated with mesogenic groups as physical crosslinker: A passive smart material with potential as viscoelastic coupling. Part II: Viscoelastic and rheological properties

Sabina Horodecka[1,2], Adam Strachota[1]*, Beata Mossety-Leszczak[3], Maciej Kisiel[3], Beata Strachota[1], Miroslav Šlouf[1]

[1)] Institute of Macromolecular Chemistry, Czech Academy of Sciences, Heyrovskeho nam. 2, CZ-162 06 Praha, Czech Republic

[2)] Faculty of Science, Charles University, Albertov 6, CZ-128 00 Praha 2, Czech Republic

[3)] Faculty of Chemistry, Rzeszow University of Technology, al. Powstancow Warszawy 6, PL-35-959 Rzeszow, Poland

1. Gelation study via T-dependent multi-frequency rheology experiments

H21–BAFKU$_2$ cooling scan

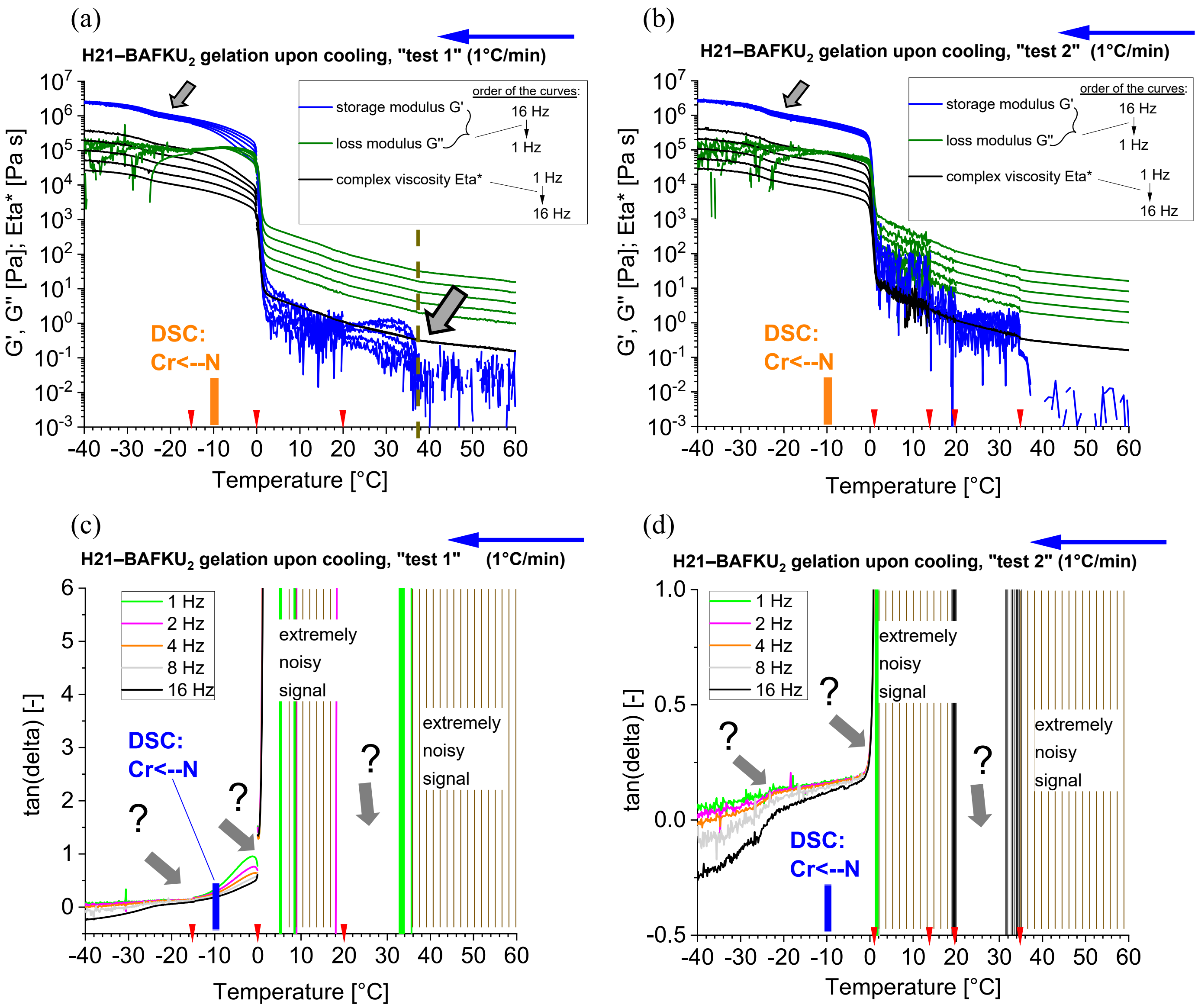

SI-Fig. 1: *Multi-frequency temperature ramp tests -* ***cooling scans*** *- carried out in the rubbery and melt temperature regions of H21–BAFKU$_2$: (a), (b): temperature dependence of the storage shear moduli G', of the loss moduli G", of the complex viscosities Eta* recorded at the simultaneously applied frequencies of 1, 2, 4, 8 and 16 Hz; (c), (d): sets of the tan δ curves with marked crossover (or near-crossover) points; (a), (c): cooling scan conducted with* ***high deformation*** *amplitudes; (b), (d): cooling scan conducted with* ***low deformation*** *amplitudes; red dotted lines indicate change in strain.*

***H21–BAFKU$_2$* heating scan**

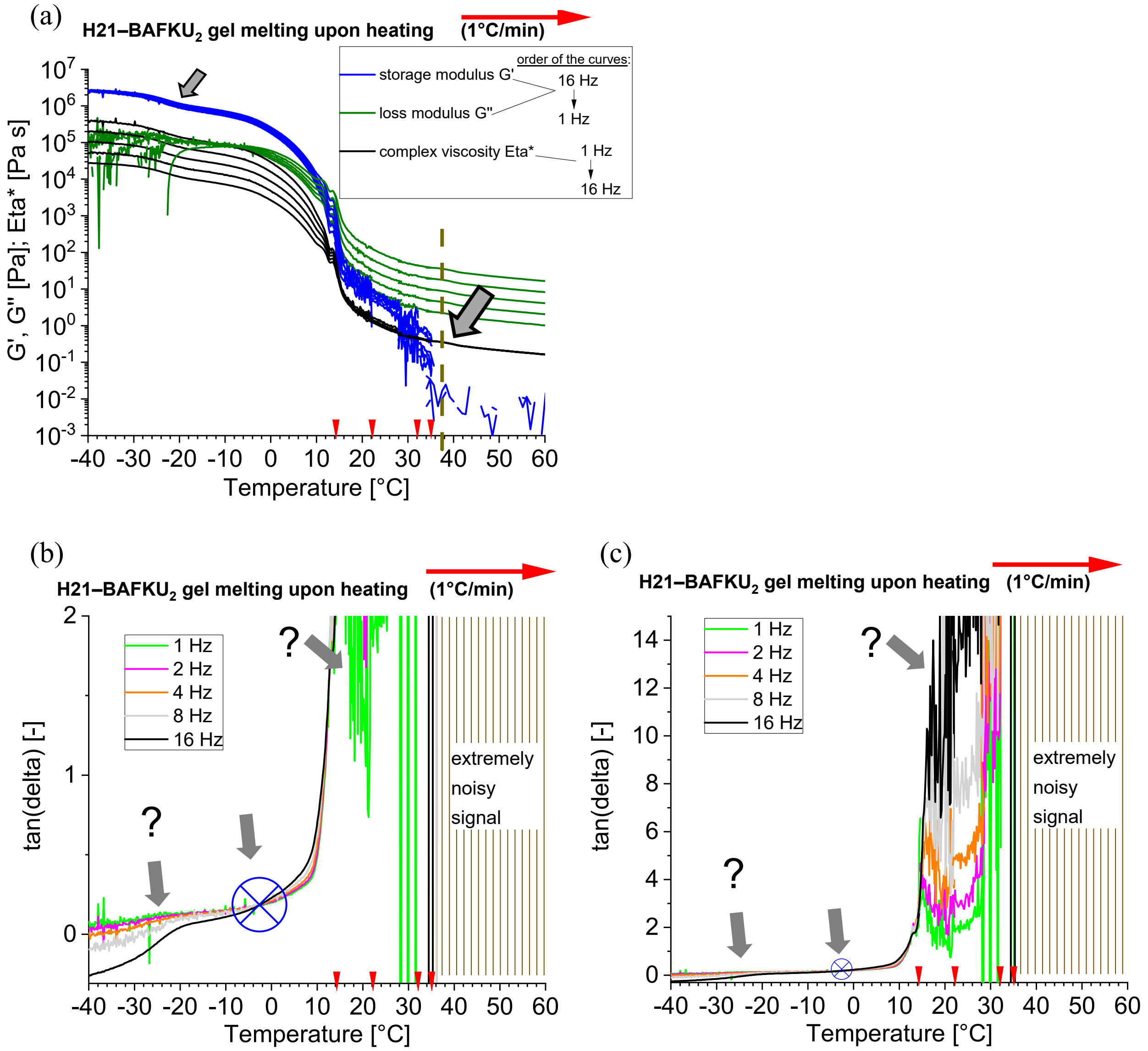

SI-Fig. 2: *Multi-frequency temperature ramp test - heating scan - carried out in the rubbery and melt temperature regions of H21–BAFKU$_2$: (a): temperature dependence of the storage shear moduli G', of the loss moduli G", of the complex viscosities Eta* recorded at the simultaneously applied frequencies of 1, 2, 4, 8 and 16 Hz as heating scan; (b): sets of the tan δ curves with marked crossover (or near-crossover) points as heating scan; (c): a different zoom of the image (b) ; red dotted lines indicate change in strain.*

2. Kinetics of gelation upon abrupt cooling of melt

<u>H11–BAFKU$_2$: all graphs</u>

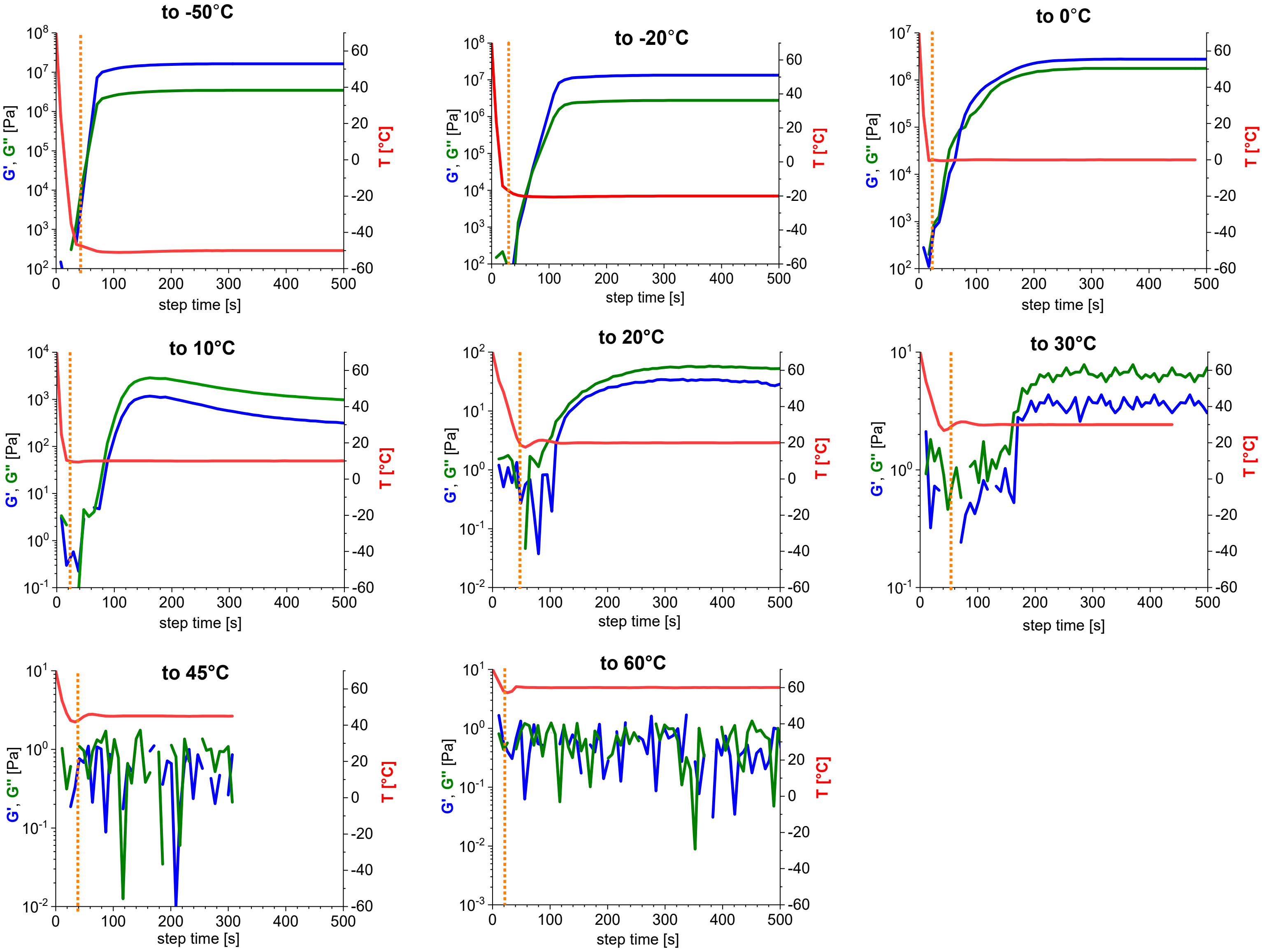

SI-Fig. 3: *Full data set:* ***Kinetics*** *of the change in storage (G') and loss (G") modulus (kinetics of physical gelation) upon* ***cooling molten H11–BAFKU$_2$*** *from 70°C down to different temperatures ranging between -50°C and +60°C; the course of the temperature of the plates between which the sample was loaded is also depicted.*

H21–BAFKU₂

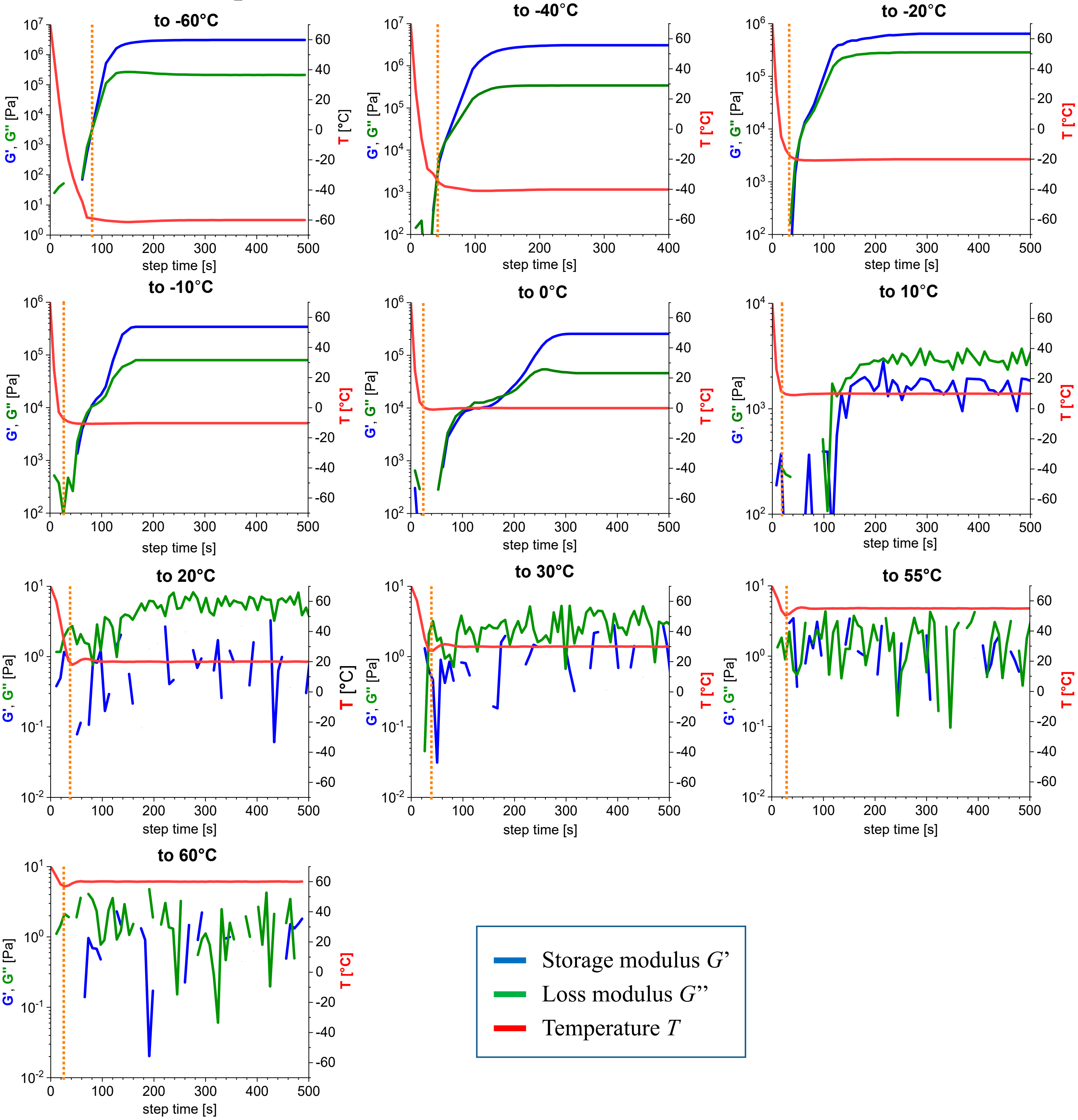

SI-Fig. 4: *Kinetics of the change in storage (G') and loss (G'') modulus (kinetics of physical gelation) upon cooling molten H21–BAFKU₂ from 70°C to different temperatures ranging between -60°C and 60°C; the course of the temperature of the plates between which the sample was loaded is also depicted.*

H03–BAFKU$_2$

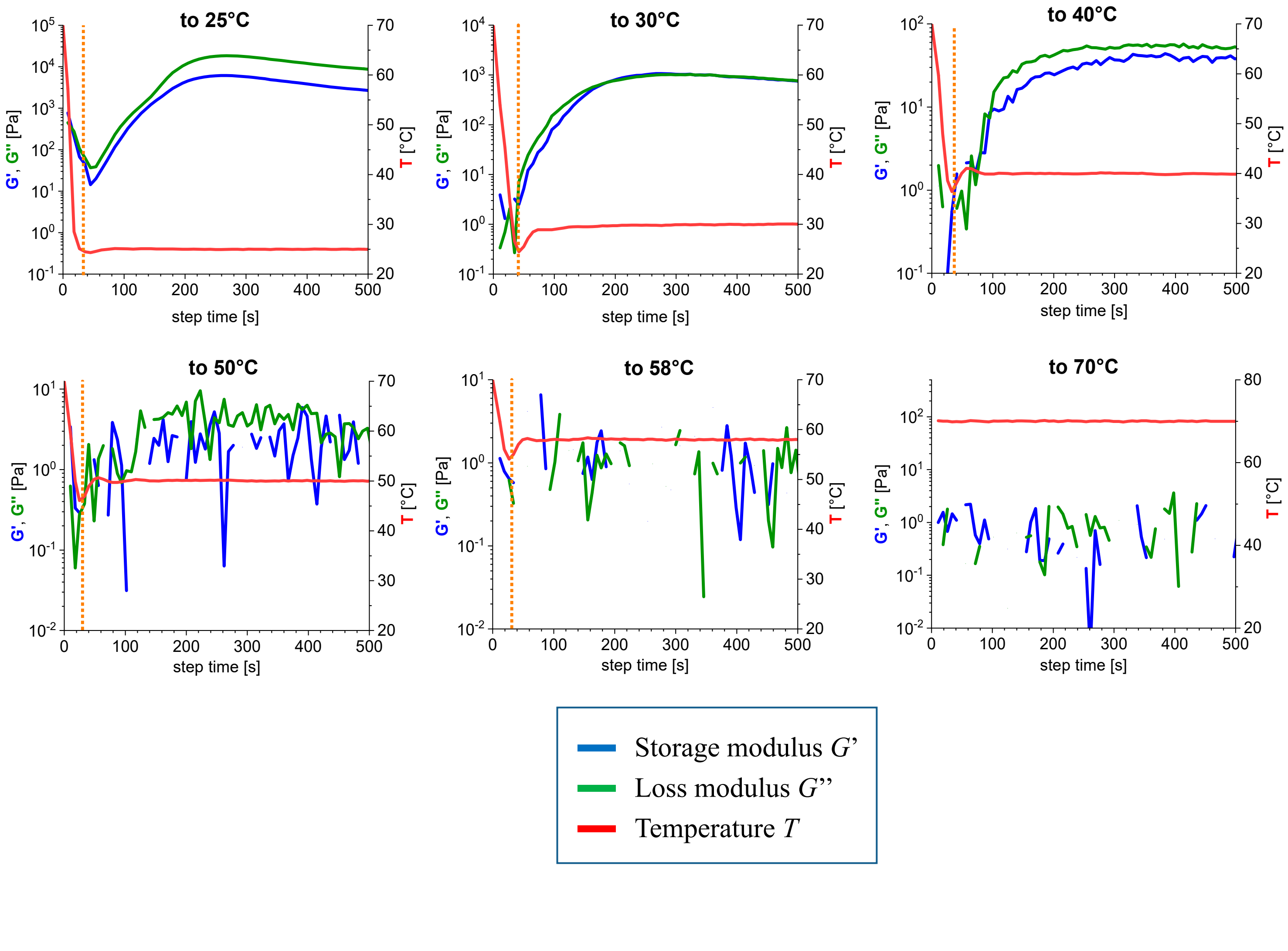

SI-Fig. 5:* *Kinetics *of the change in storage (G') and loss (G'') modulus (kinetics of physical gelation) upon* ***cooling molten H03–BAFKU$_2$*** *from 70°C down to different temperatures ranging between +30°C and +70°C; the course of the temperature of the plates between which the sample was loaded is also depicted.*

3. Disconnection of the physical network by high mechanical strain

H11–BAFKU₂

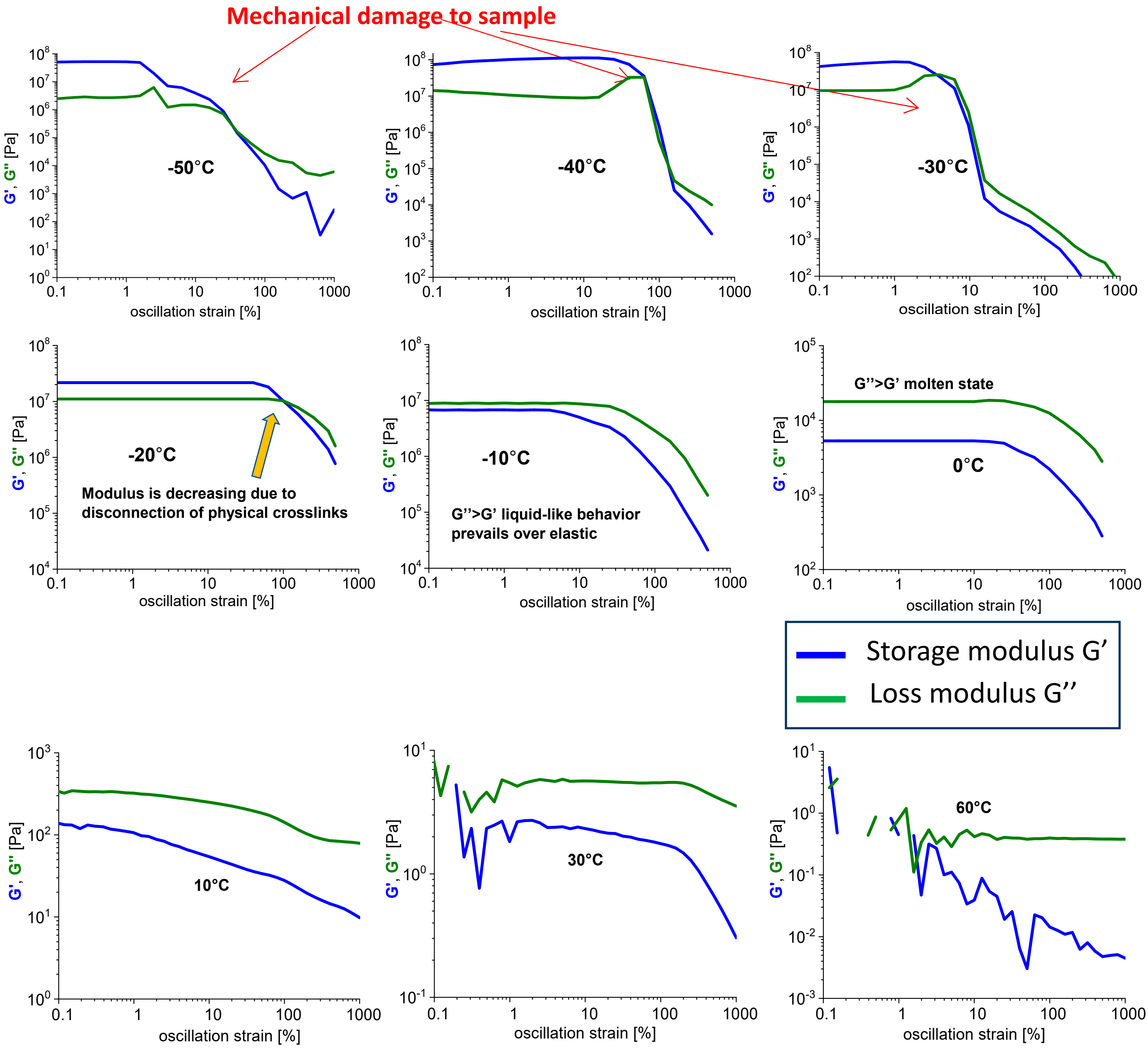

SI-Fig. 6: *All data: Disconnection of the physical crosslinks in H11–BAFKU₂ by mechanical strain: strain-dependence of storage (G') and loss (G'') modulus of H21–BAFKU₂ at the temperatures from -50 to +60°C.*

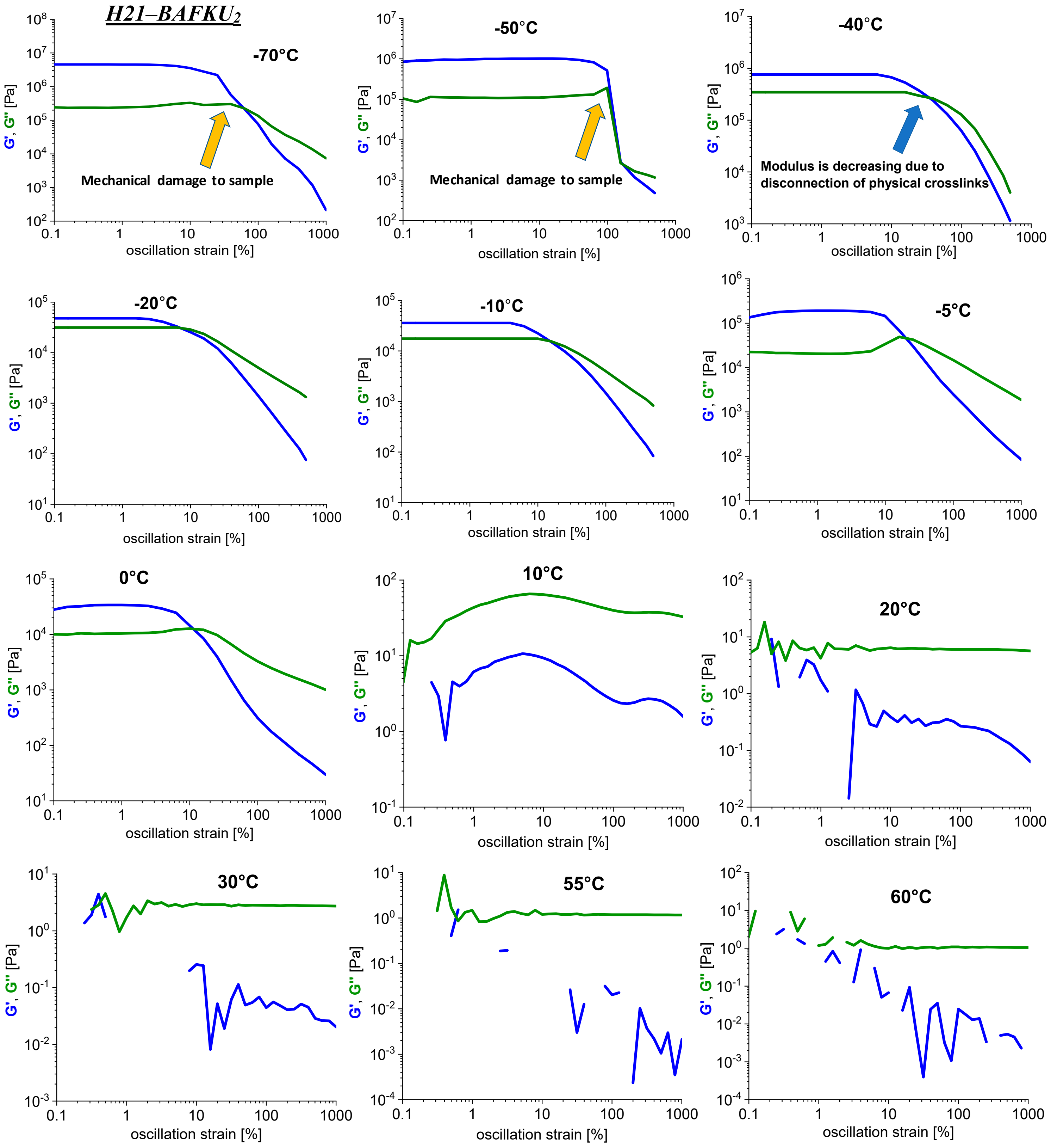

***SI-Fig. 7:** Disconnection of the physical crosslinks in H21–BAFKU$_2$ by mechanical strain: strain-dependence of storage (G') and loss (G'') modulus of H21–BAFKU$_2$ at the temperatures from -70 to +60°C.*

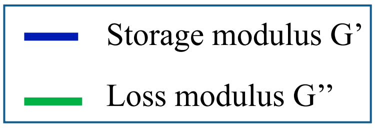

H03–BAFKU$_2$

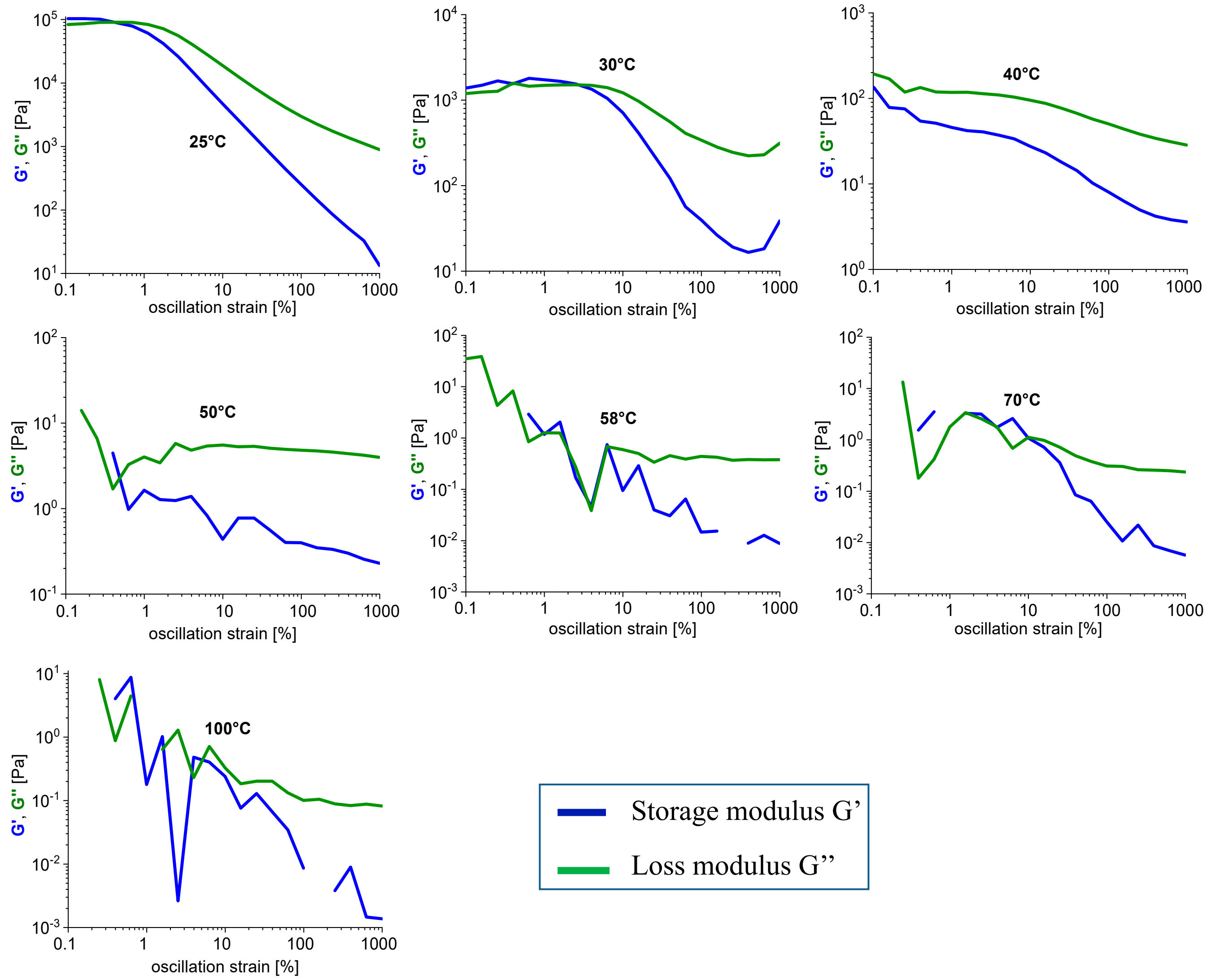

SI-Fig. 8: *Disconnection of residual physical crosslinks in H03–BAFKU$_2$* melt *by mechanical strain: strain-dependence of storage (G’) and loss (G”) modulus of H03-BAFKU$_2$ at the temperatures from +25 to +100°C.*

Creep tests

H11-BAFKU2 all data

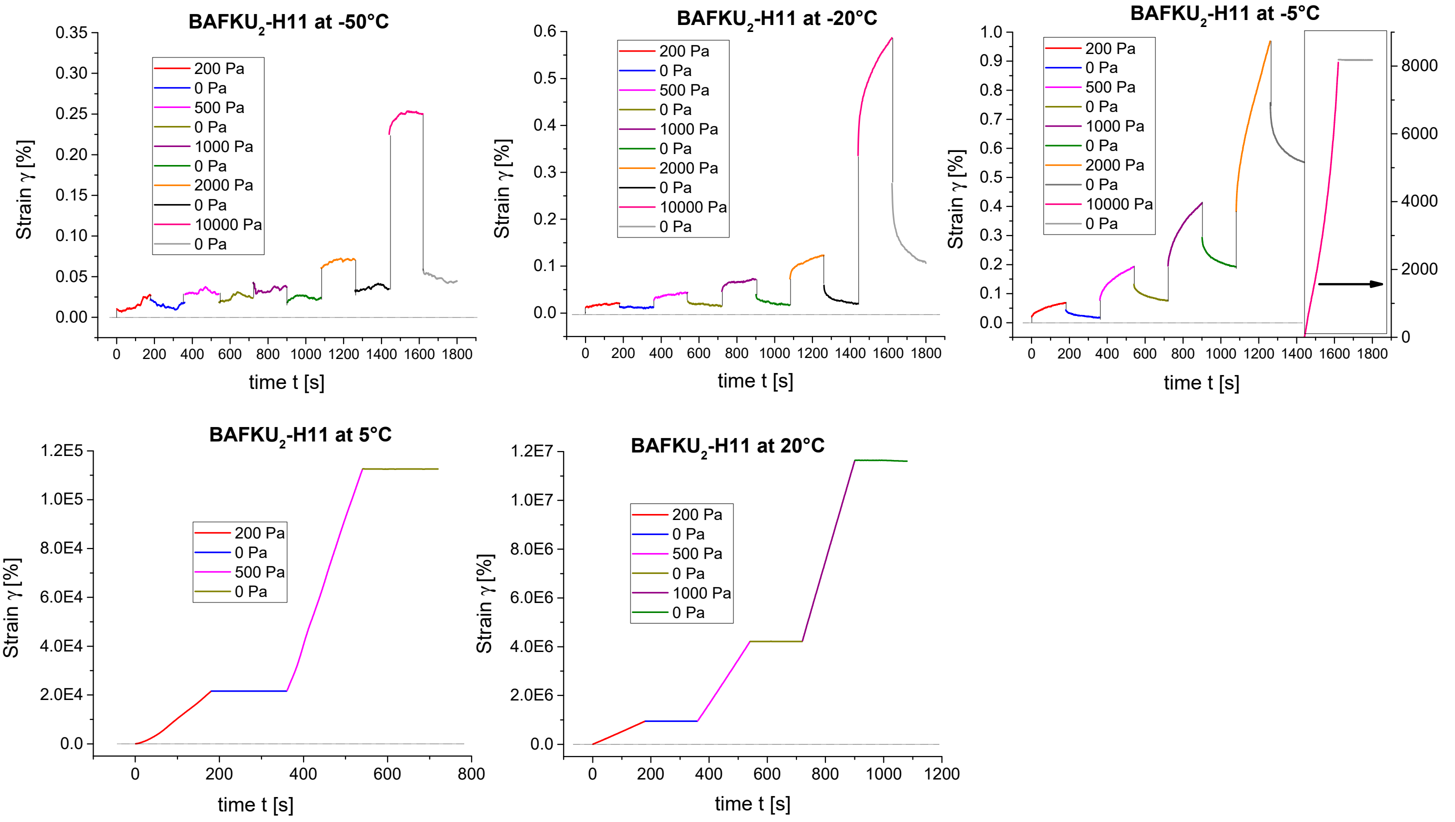

SI-Fig. 9: *Multi-step creep tests of H11–BAFKU$_2$ all data: tests at temperatures between -50 and +20°C; stresses ranging between 200 and 10 000 Pa were applied, followed by recovery steps (at 0 Pa).*

H21-BAFKU2

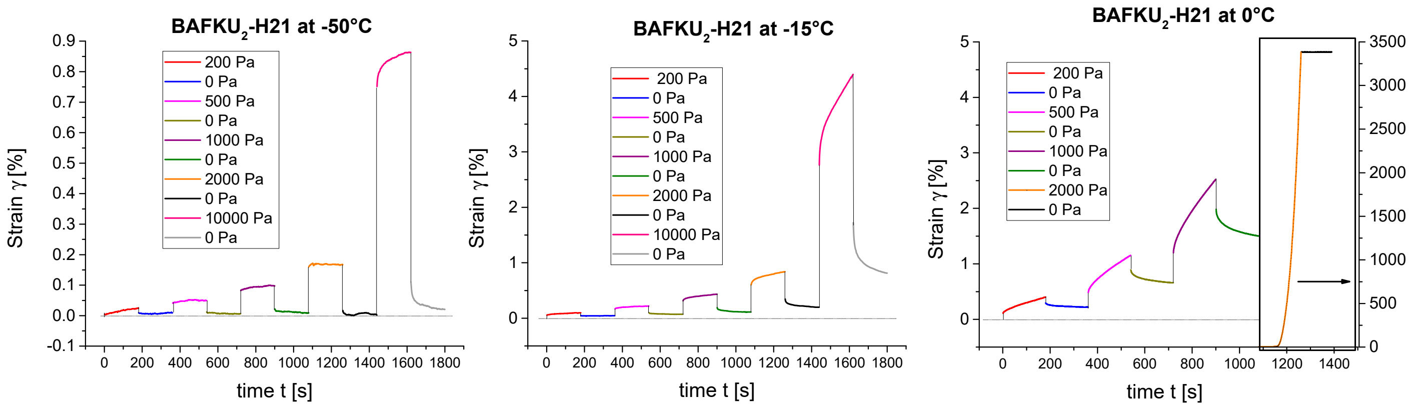

SI-Fig. 10: *Multi-step creep tests of H21–BAFKU$_2$ at temperatures between -50 and 0°C; stresses ranging between 200 and 10 000 Pa were applied, followed by recovery steps (at 0 Pa).*

H03-BAFKU2

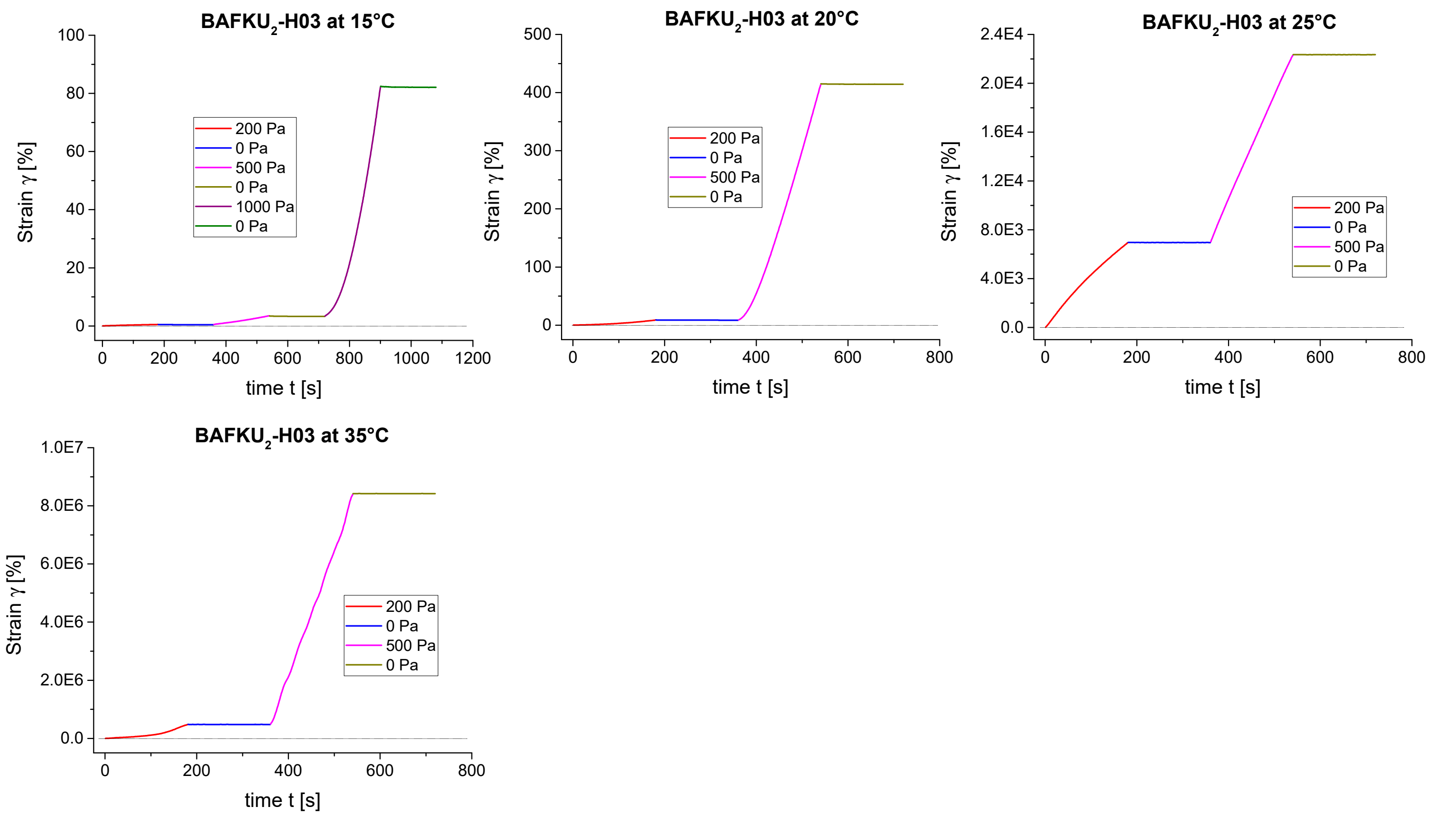

SI-Fig. 11: *Multi-step creep tests of H03–BAFKU2 at temperatures between +15 and +35°C; stresses ranging between 200 and 1 000 Pa were applied, followed by recovery steps (at 0 Pa).*

Relaxation tests

H11-BAFKU2

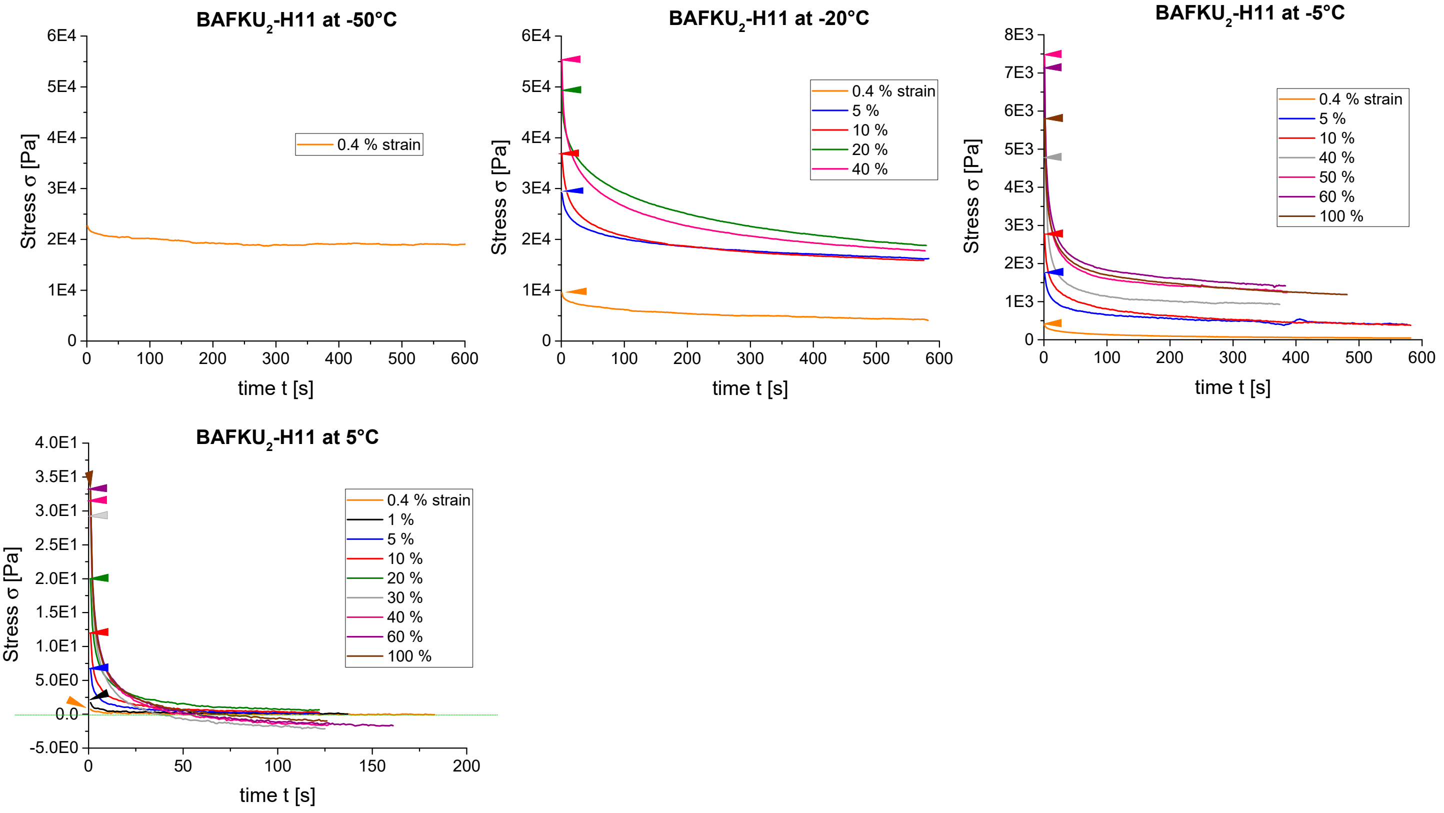

SI-Fig. 12: *Stress relaxation tests of H11–BAFKU*$_2$ *at temperatures between -50 and +5°C; at each temperature, several constant strain values were applied.*

Relaxation H03-BAFKU2

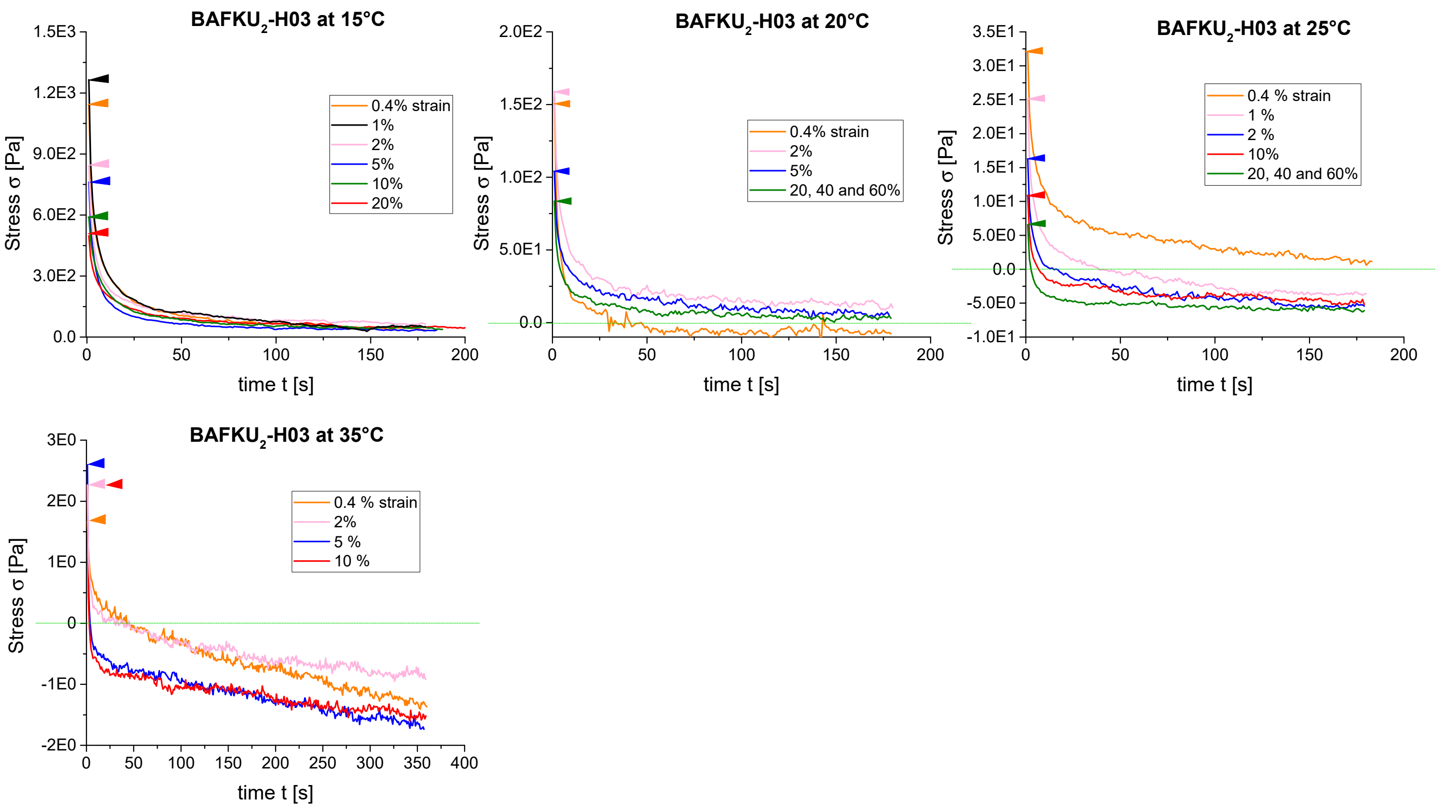

SI-Fig. 13: *Stress relaxation tests of H03–BAFKU2 at temperatures between between +15 and +35°C; at each temperature, several constant strain values were applied.*

4. High-frequency stiffening and self-healing effects

H11–BAFKU$_2$

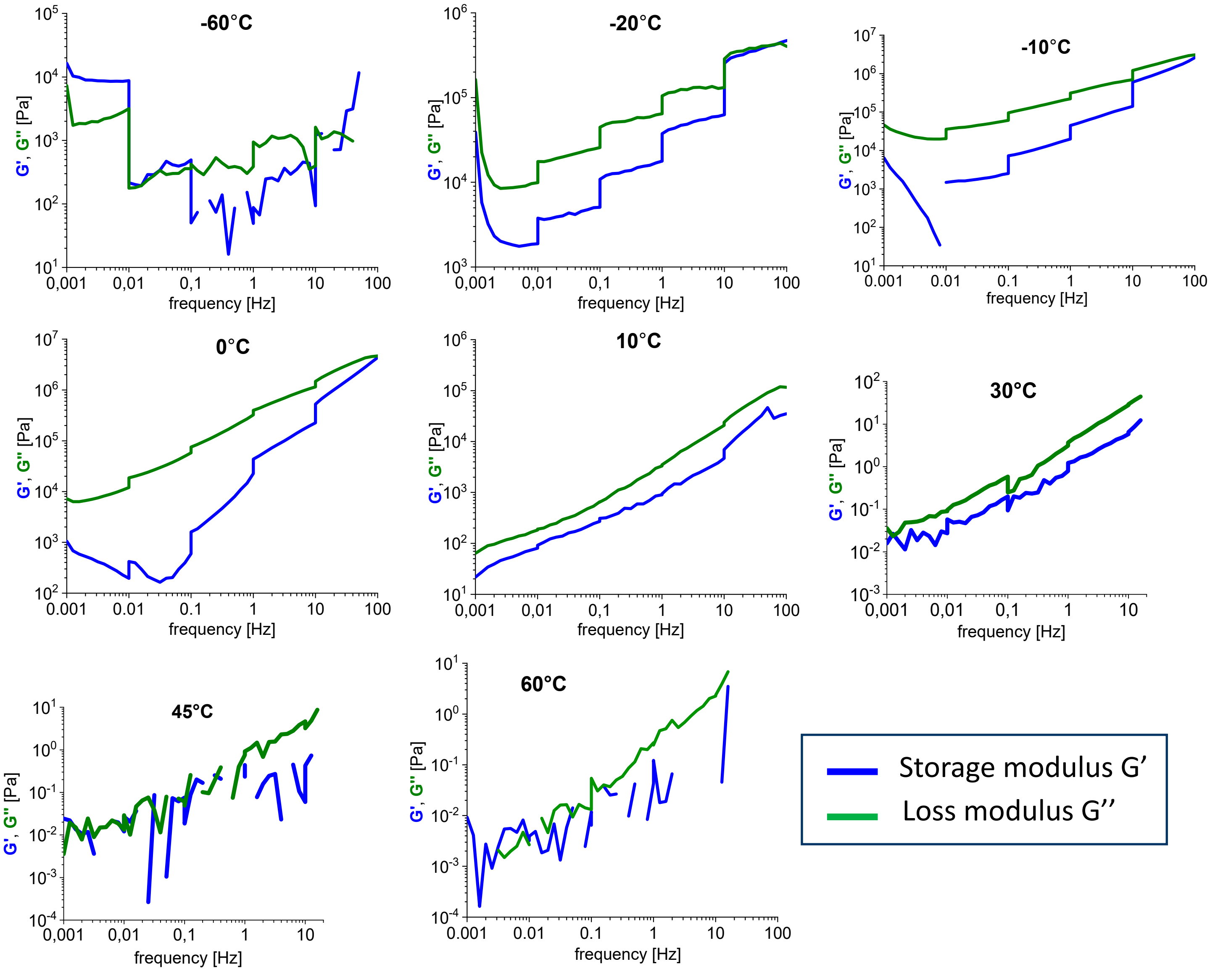

SI-Fig. 14: *All data: Frequency-stiffening of H11–BAFKU$_2$ observed in frequency sweep tests (1 mHz to 100 Hz) conducted at temperatures between -60 and +60°C; the strain amplitude was different in each frequency decade, ranging from 50% at 1 mHz to 1% at 100 Hz; the effect of strain-induced damage to the network, as well as of its recovery between the frequency decades is clearly visible, especially in case of G’ curves at lower temperatures.*

H21-BAFKU$_2$

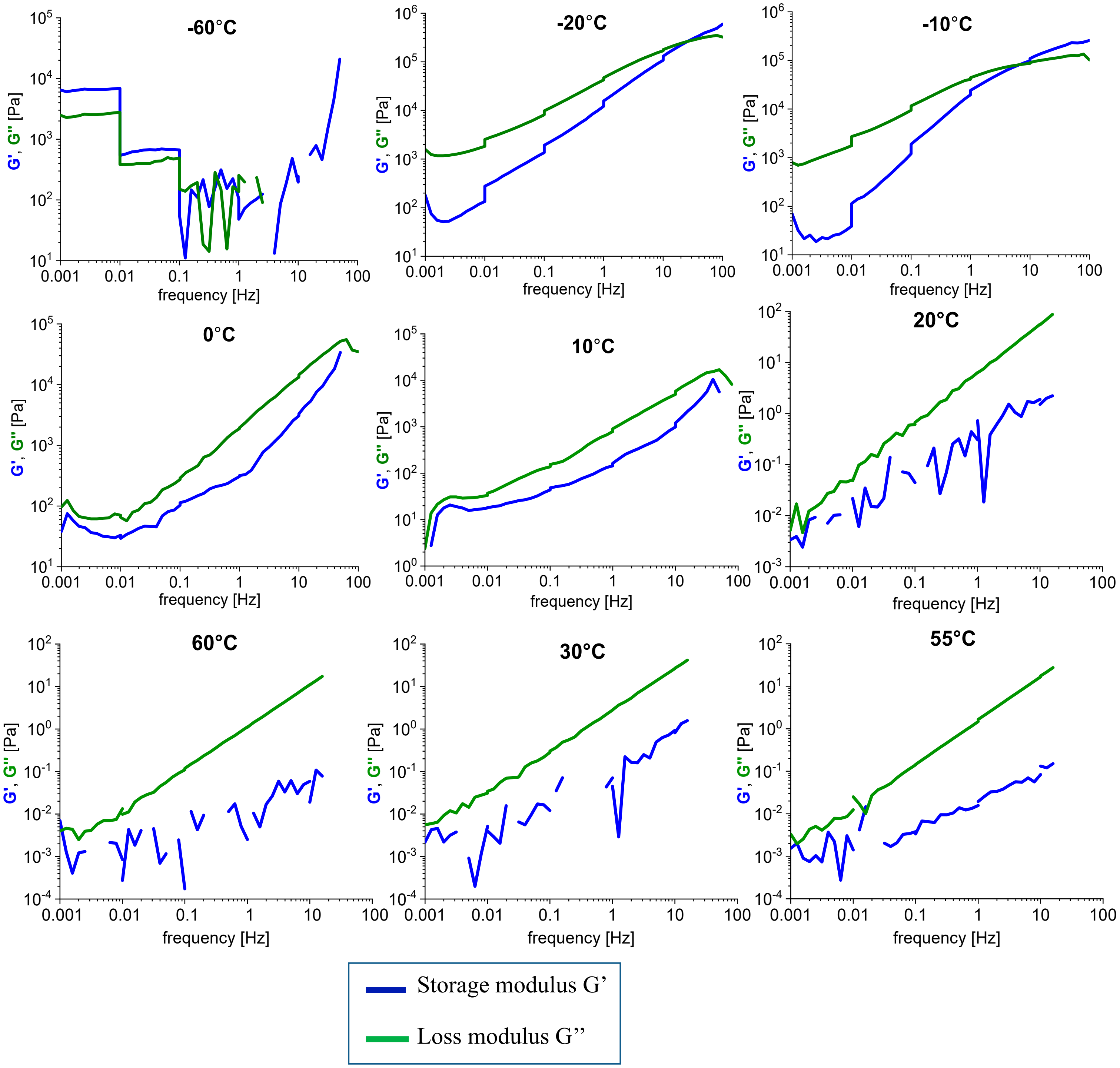

SI-Fig. 15: *Frequency-stiffening of H21–BAFKU$_2$ observed in frequency sweep tests (1 mHz to 100 Hz) conducted between -60 and +55°C; the strain amplitude was different in each frequency decade, ranging from 50% at 1 mHz to 1% at 100 Hz; the effect of strain-induced damage to the network, as well as of its recovery between the frequency decades is clearly visible, especially in case of G' curves at lower temperatures.*

H03-BAFKU$_2$

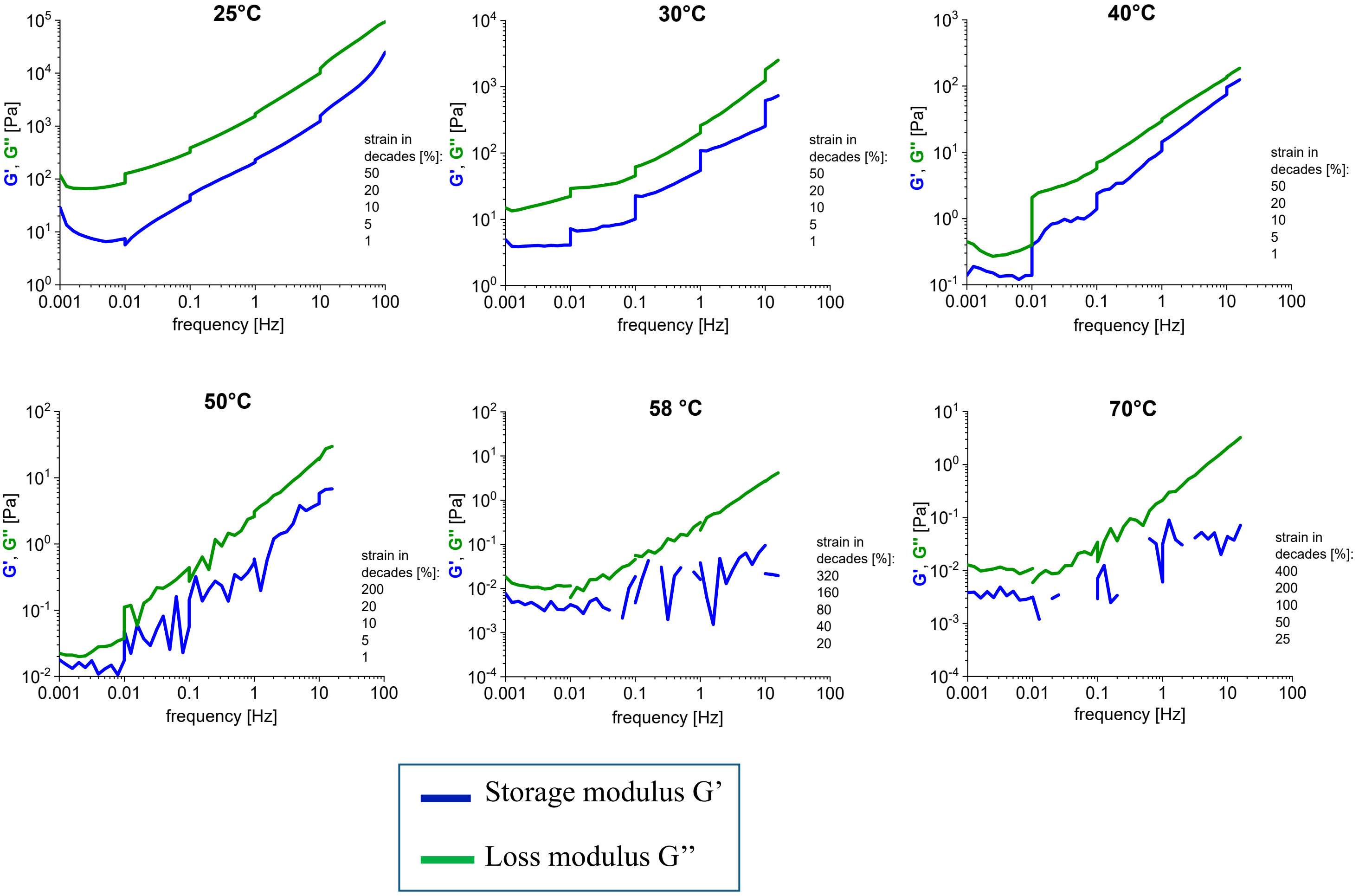

SI-Fig. 16: *Frequency-stiffening of H03–BAFKU$_2$ melt observed in frequency sweep tests (1 mHz to 100 Hz) conducted at temperatures between +25 and +70°C; the strain amplitude was different in each frequency decade, ranging from 50% at 1 mHz to 1% at 100 Hz; the effect of strain-induced damage to elastic structures in the melt, as well as their recovery between the frequency decades is clearly visible, especially at lower temperatures.*

Explanation of the upward steps in G”

While simple self-healing should lead to decrease in *G*” (stronger elastomer character due to more crosslinks, shorter elastic chains and hence less friction), the experimentally observed upward steps in *G*” could be explained by resistance caused by re-assembled larger aggregates (lamellae) of BAFKU, which have time to disconnect at lower frequencies (and high applied strains). Their gradual destruction by shear generates resistance (high *G*” value), but also leads to a decrease in the number of these secondary aggregates and thus in turn to less than maximum resistance (smaller growth, or in extreme cases even local decrease in *G*”). During the experimental delay, the smaller BAFKU aggregates (‘fragments’) re-assemble to larger ones again, and hence can generate considerably increased resistance after the delay (upward step in G”).

5. Thixotropy effects

H03-BAFKU$_2$ kinetics of gelation upon cooling

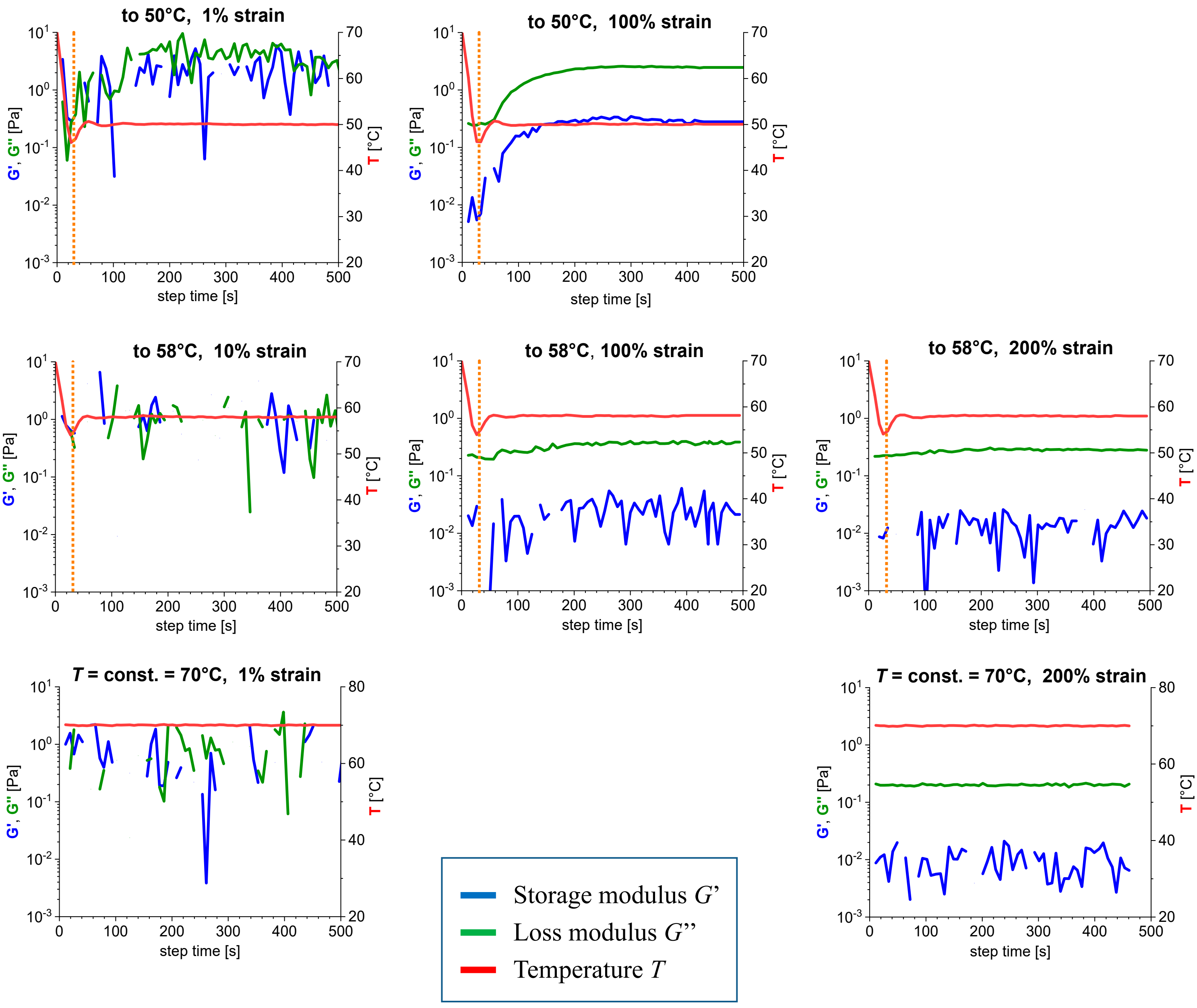

SI-Fig. 17: *Thixotropy effects visible as strong strain-dependence of the moduli values (G', G'') in case of the kinetics of physical gelation of molten H03–BAFKU$_2$ upon abrupt cooling.*

Very small strains also lead to change in measured moduli:

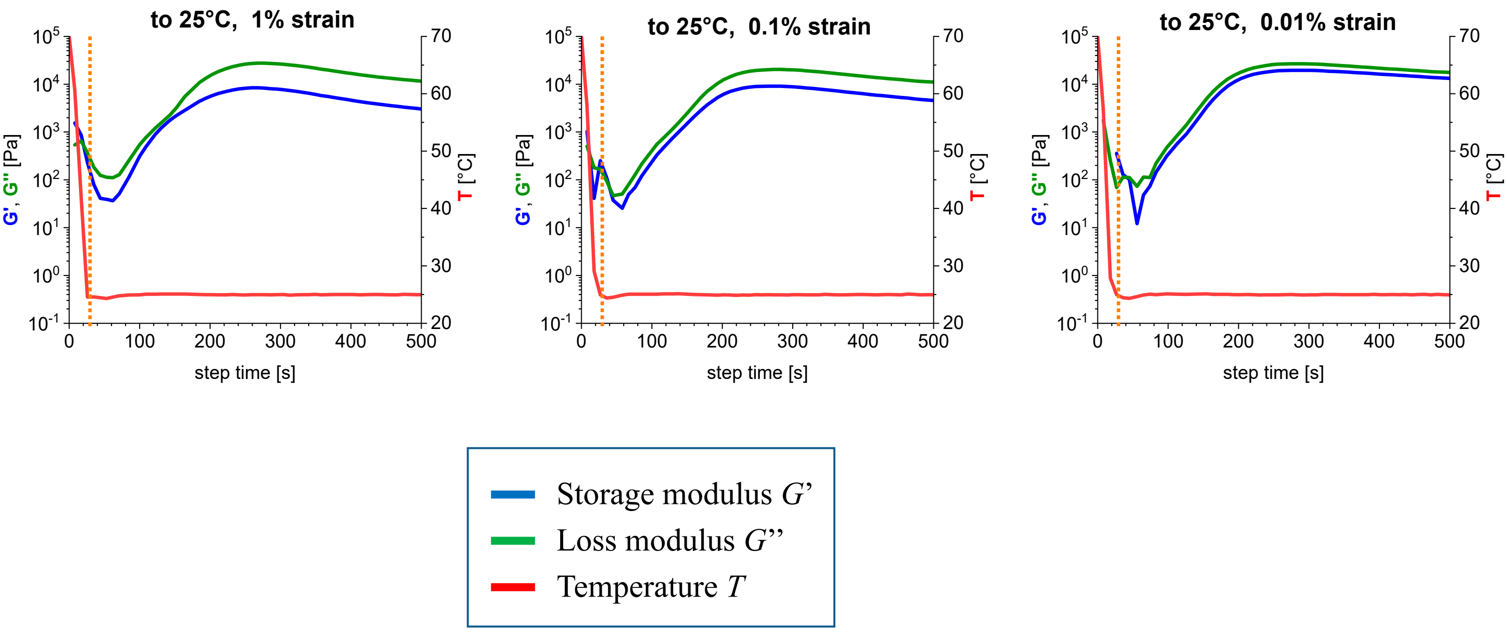

SI-Fig. 18: *Thixotropy effects as strain-dependence – study of very low strains – of the moduli values and curve course – especially G', less so G'', in case of the kinetics of physical gelation of molten H03–BAFKU*$_2$ *upon abrupt cooling.*

H11-BAFKU$_2$ kinetics of gelation upon cooling

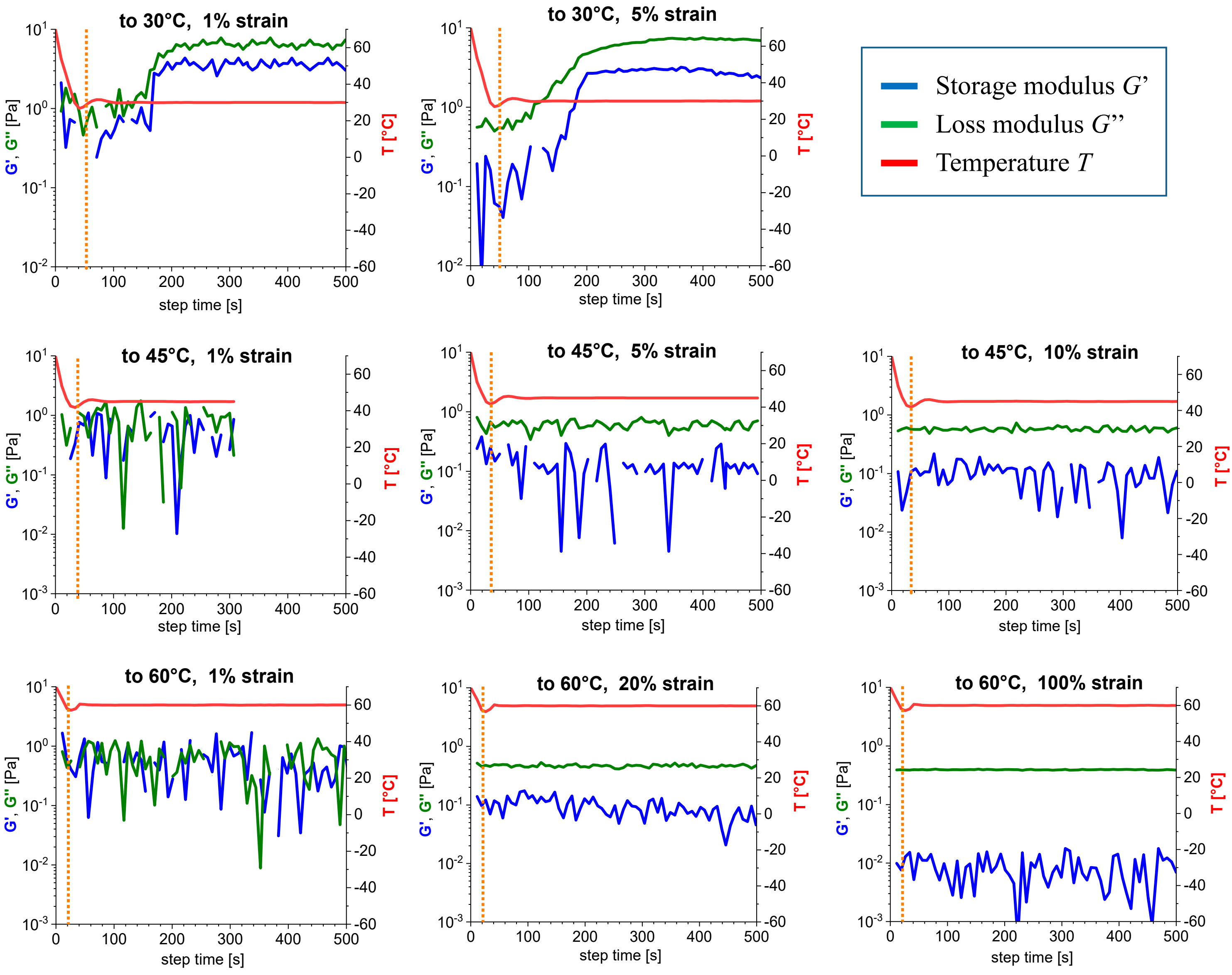

SI-Fig. 19: *Thixotropy effects visible as strong strain-dependence of the moduli values (G', G'') in case of the kinetics of physical gelation of molten H11–BAFKU$_2$ upon abrupt cooling.*

H21-BAFKU$_2$ kinetics of gelation upon cooling

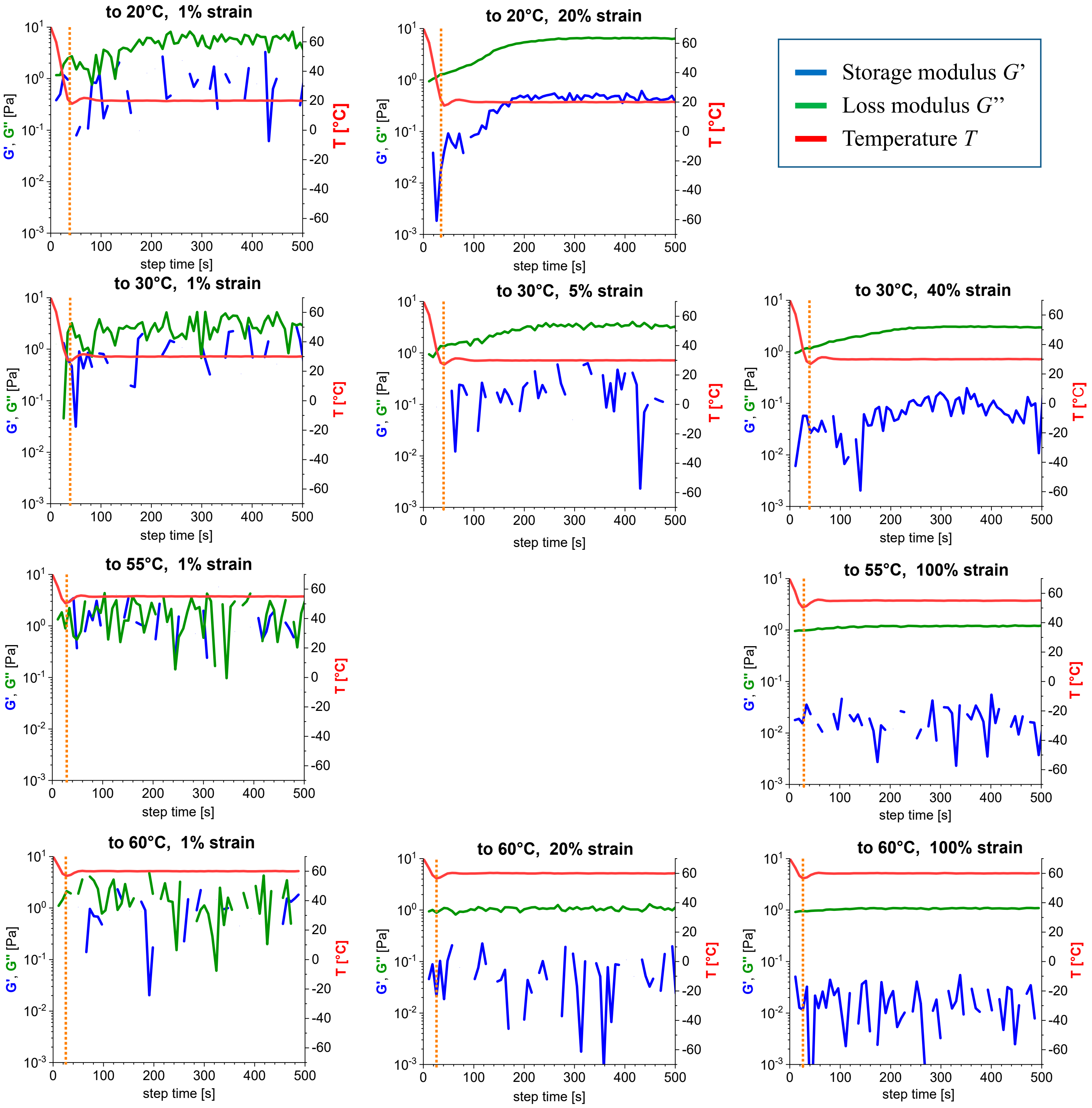

***SI-Fig. 20:** Thixotropy effects visible as strong strain-dependence of the moduli values (G', G'') in case of the kinetics of physical gelation of molten H21–BAFKU$_2$ upon abrupt cooling.*

Thixotropic Loop tests

H21-BAFKU2

0°C

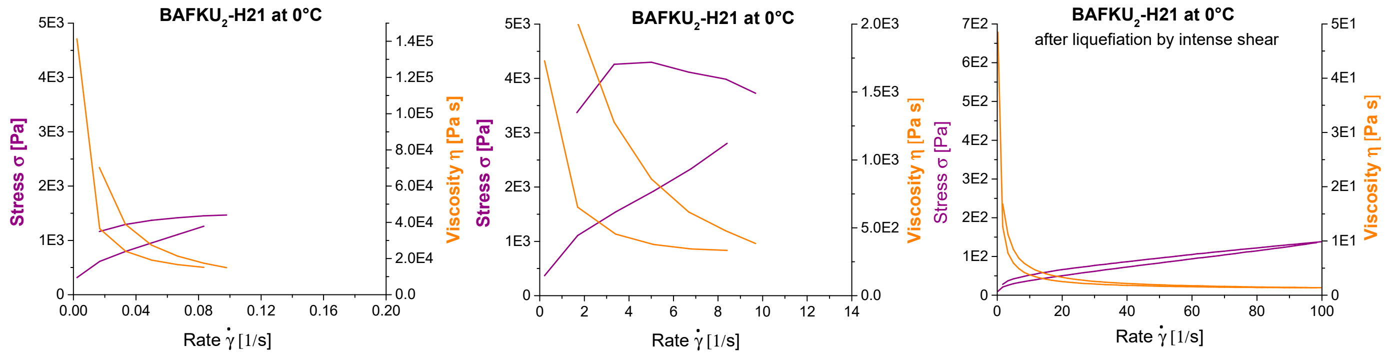

20°C

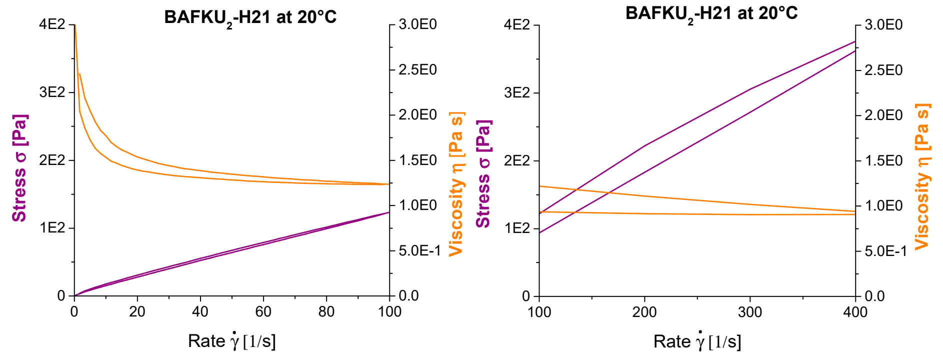

80°C

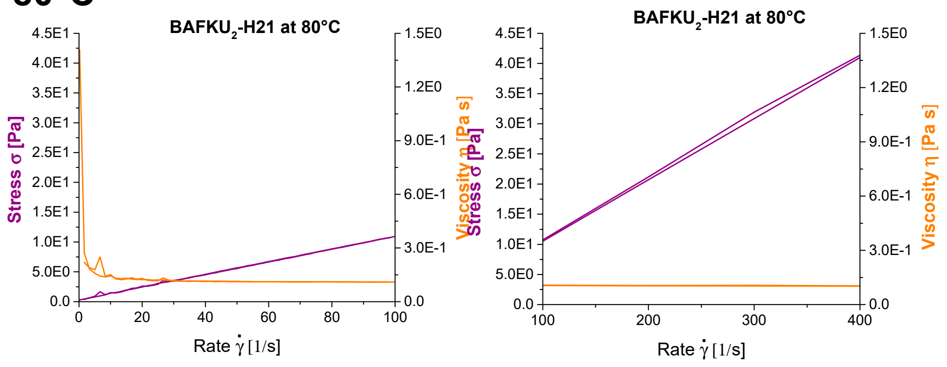

SI-Fig. 21: *Thixotropic loop tests (dependence of shear stress and of viscosity on the shear rate) for the copolymer H21–BAFKU2 at 0, 20 and 80°C: at the left are continuous tests with shear rates rising up to 100* s^{-1}*, at the right are step-wise tests with shear rates between 100 and 400* s^{-1}*.*

H03-BAFKU2

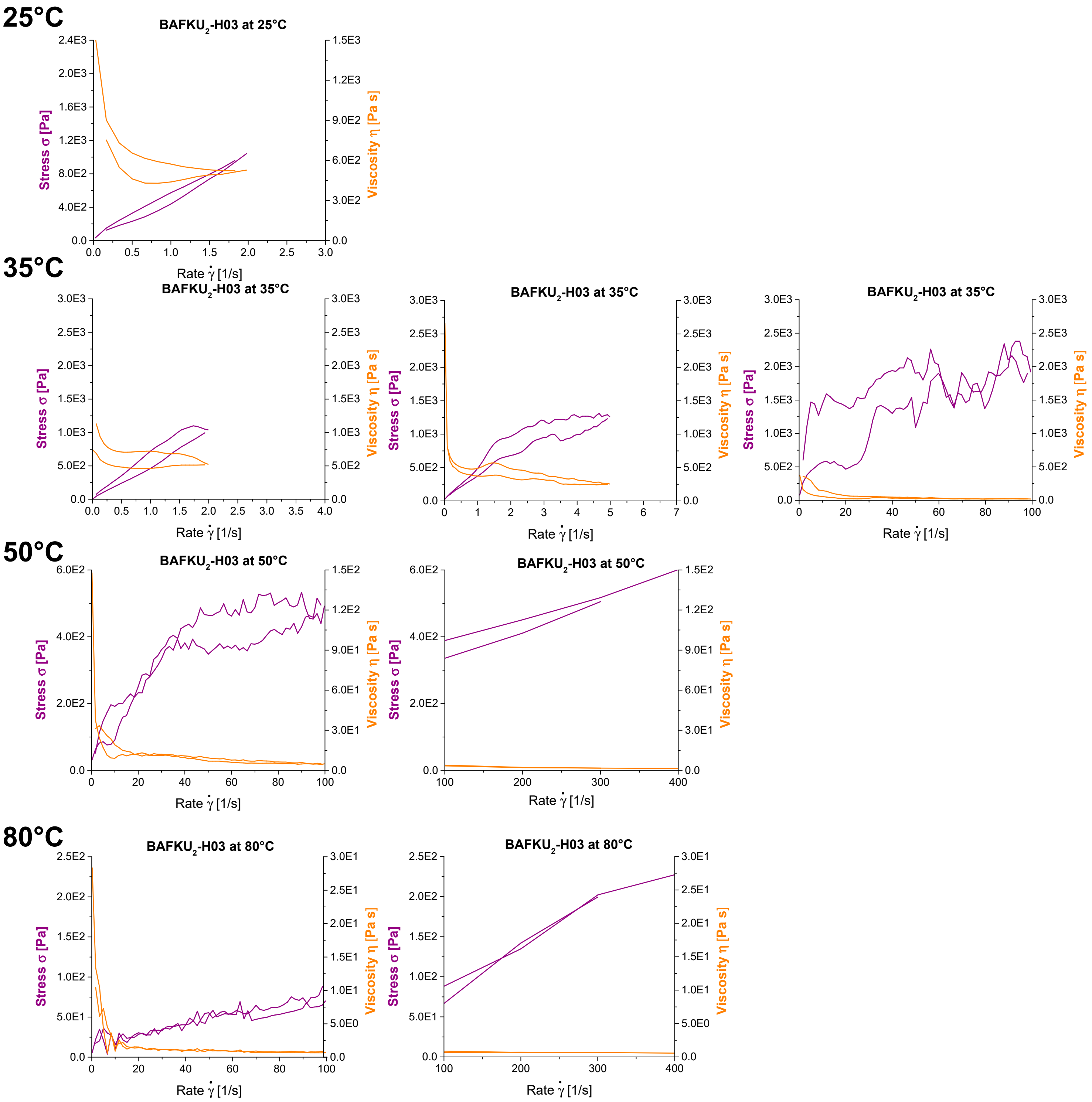

SI-Fig. 22: *Thixotropic loop tests (dependence of shear stress and of viscosity on the shear rate) for the copolymer H03–BAFKU2 at 25, 35, 50 and 80°C: at the left are continuous tests with shear rates rising up to 100 s^{-1}, at the right are step-wise tests with shear rates between 100 and 400 s^{-1}; at 35°C, only 100 s^{-1}could be reached, but two low-shear-rates tests were performed.*
